# Meta-analysis of coefficient alpha for scores on the Narcissistic Personality Inventory

**DOI:** 10.1371/journal.pone.0208331

**Published:** 2018-12-04

**Authors:** Brian K. Miller, Kay M. Nicols, Silvia Clark, Alison Daniels, Whitney Grant

**Affiliations:** 1 Department of Management, Texas State University, San Marcos, Texas, United States of America; 2 Department of Management, University of South Carolina, Columbia, South Carolina, United States of America; Tilburg University, NETHERLANDS

## Abstract

The Narcissistic Personality Inventory (NPI) has greatly facilitated the scientific study of trait narcissism. However, there is great variability in the reported reliability of scores on the NPI. This study meta-analyzes coefficient alpha for scores on the NPI and its sub-scales (e.g. entitlement) with transformed alphas weighted by the inverse of the variance of alpha. Three coders evaluated 1213 individual studies for possible inclusion and determined that 1122 independent samples were suitable for coding on 12 different characteristics of the sample, scale, and study. A fourth author cross-coded 15 percent of these samples resulting in 85 percent overall agreement. In the independent samples, comprised of 195,038 self-reports, the expected population coefficient alpha for the NPI was .82. The population value for alpha on the various sub-scales ranged from .48 for narcissistic self-sufficiency to .76 for narcissistic leadership/authority. Because significant heterogeneity existed in coded study alphas for the overall NPI, moderator tests and an explanatory model were also conducted and reported. It was found that longer scales, the use of a Likert response scale as opposed to the original forced choice response format, higher mean scores and larger standard deviations on the scale, as well as the use of samples with a larger percentage of female respondents were all positively related to the expected population alpha for scores on the overall NPI. These results will likely aid researchers who are concerned with the reliability of scores on the NPI in their research on non-clinical subjects.

## Introduction

Narcissism is a particularly insidious personality trait characterized by grandiosity, self-absorption, and a lack of empathy that can make life difficult for the narcissist [1] as well as for others [2,3]. The scientific study of sub-clinical narcissism was greatly aided by the development of the Narcissistic Personality Inventory (NPI) [4] which is the most commonly used narcissism instrument available [5]. The NPI was originally based on the diagnostic criteria for Narcissistic Personality Disorder (NPD), which was first added as a clinical disorder to the third edition of the Diagnostic and Statistical Manual in 1980 (DSM III) [6] on Axis II. Because the NPI is based on clinical diagnostic criteria, the boundary between the measurement of sub-clinical narcissism with the NPI and diagnosis of clinical NPD remains unclear [7] as the two may be parts of the same spectrum.

Despite refinements and revisions to the NPI [8,9,10,11] concerns about its psychometric properties are mounting [12] largely because of an indeterminate factor structure with between four and seven factors typically arising in multiple versions of the instrument. More recent research [13] has found a three-factor structure to scores on the inventory in four independent samples. Ongoing problems with the factor structure have led to efforts to shorten the NPI [14] and to develop alternative measures such as the Pathological Narcissism Inventory [15]. Because it measures multiple components of narcissism, the NPI is not a unidimensional scale and combining adaptive components of narcissism with its maladaptive components can be problematic. Because of this, it has been recommended that the NPI sub-scale scores should not be summed for an overall measure of narcissism [13] but there is some evidence that the sub-scales as stand-alone measures may indeed be unidimensional [16].

Partially subsumed under the issues of factor indeterminacy are problems with the reliability of scores on the NPI and its sub-scales which have all too often been exceedingly poor [12]. The reliability of scores on the overall NPI have ranged widely from a low of .61 [17] to a high of .92 [18]. Researchers are increasingly recommending the use of the sub-scales of the NPI to predict narrowly defined specific criteria rather than using the overall NPI [10,19,20,21] but scores on the sub-scales have been even less reliable than on the overall instrument. Despite this, researchers [13] have found that the narcissistic leadership/authority sub-scale is strongly positively related to the Big Five trait of extraversion, moderately negatively related to agreeableness, and unrelated to the other Big Five traits. The NPI sub-scale of grandiose exhibitionism shows similar relationships to the Big Five. However, the sub-scale of entitlement/exploitativeness is unrelated to extraversion, strongly negatively related to agreeableness, moderately negatively related to conscientiousness and openness-to-experience, and moderately positively related to neuroticism. Nevertheless, most researchers use the full-length NPI to aggregate upward to the scale level instead of examining the sub-scales as stand-alone measures. This may be problematic as discussed below in the overview of narcissism.

This study uses a refinement to the reliability generalization (RG) technique [22] to determine the expected population coefficient alpha for scores on the NPI and its sub-scales [23]. The calculation of an estimated population value of coefficient alpha is helpful in the determination of whether the reliability of scores on the NPI and its sub-scales meet the standards for proper usage.

### Overview of narcissism

Narcissism is based upon Ovid’s retelling [24] of the Greco-Roman fable of Narcissus who spurned the love of the nymph Echo and instead fell in love with his own reflection in a pool of water. Being unable to avert his loving gaze he withered away and died. Given the tragedy of such self-love, narcissism is typically viewed negatively, but can have some adaptive components [25] like authority and leadership. Despite the negative connotations associated with narcissism, its adaptive components are still important to the overall picture of a narcissist [26,27] but most research focuses on the maladaptive aspects of narcissism like entitlement, exploitativeness, superiority, exhibitionism, and vanity. Despite well-known problems with summing the sub-scales of the NPI it is the most ubiquitously used [5] measure of overall narcissism. Analyses with it have provided some evidence that the level of trait narcissism in the general public is on the rise [28,29], as well as a few dissenting views [30,31] both of which have helped spur an interest in narcissism amongst academics, the popular press, and lay persons.

Recently the diagnosis of NPD was updated in the fifth edition of the Diagnostic and Statistical Manual (DSM-5)[32] which also suggested a trait-based dimensional model for the diagnosis of personality disorders. Researchers have long advocated the trait-based dimensional measurement of various personality disorders [33,34] which made its way into the DSM-5 and by now well over 100 such studies have used this new method to diagnose NPD [35]. These trait-based models often use facets of the Five Factor Model of personality. Despite no longer being useful for NPD diagnosis, the importance of the reliability of scores on the NPI as a measure of sub-clinical narcissism cannot be overstated.

### Reliability generalization

Not so long ago, researchers were admonished to report the reliability coefficient for scores in their own samples and stop inferring reliability from coefficients published in other sources [36]. This is known as reliability induction [37] and is the bane of the psychometrician conducting an RG study. Reliability generalization began as a technique designed to determine the study, scale, and sample characteristics associated with the reliability of scores on an instrument [23]. Examples include RG analyses of the Speilberger State-Trait Anxiety Inventory [38], two locus of control scales [39], the Marlowe-Crowne Social Desirability Scale [40], and the Life Satisfaction Index [41]. Studies like these tend to use ordinary least squares regression to ascertain the unique variance in a set of reliability coefficients attributed to numerous coded study characteristics. However, this technique often suffers because of the use of listwise deletion inherent to regression that arises when not all primary studies report all coded characteristics.

Recent refinements to the RG technique [23] have built upon other statistical techniques [42] that seek to meta-analyze coefficient alpha with the purpose of estimating the population value for alpha from a sample of studies. This technique first transforms the usually non-normally distributed alpha and then weights each transformed alpha by the inverse of the variance of alpha. Alpha is only an approximation of the reliability of scores on an instrument in a sample but the variance of the distribution of alpha in a sample can be calculated [43] and used to establish confidence intervals around the point estimate for alpha for that sample. Using the inverse of the variance of alpha as a weighting mechanism gives greater weight to alpha coefficients that have smaller variances for the reliability of scores in each sample. That is, more accurately estimated alpha coefficients get greater weight in the meta-analysis of coefficient alpha. This technique [23] has been used in reliability studies of the Yale-Brown Obsessive Compulsive Scale for adults [44] as well as for children and adolescents [45] and for the Physical Self-Description Questionnaire [46]. Recently, a variation of this technique was applied to studies gathered from published validity generalizations of various personality traits in five top journals [47]. In that study the population value of alpha on a variety of different measures of narcissism was .83. However, that analysis used untransformed alpha, made no attempt at obtaining all published studies on narcissism, used a wide variety of measures of narcissism, did not calculate the population alpha for the sub-scales of any measure of narcissism, and made use of a greatly reduced sample (both studies and subjects) size.

The purpose of the current study was to use this technique [23] to calculate the population reliability coefficient for scores specifically on the NPI using all extant published studies. This required a thorough search of several databases for primary studies and determine whether they were appropriate for inclusion, code the appropriate studies, cross-code some of them to determine interrater agreement, and apply Eqs 1 through 6 below [23] to the transformed reliability coefficients as described below. Our focus, as in most RG studies using this technique [23] was on one scale only: the NPI.

## Methods

### Data sources

For inclusion in the meta-analysis, a study had to meet four criteria. It had to be: (1) a peer-reviewed published study, (2) report a reliability coefficient for self-reported scores on the overall NPI or any of its sub-scales in one or more independent samples, (3) gather scores using adult participants (age ≥ 18 years), and (4) be published in English. Our exclusive reliance on published articles may have introduced some publication bias in that unpublished research may suffer from low reliability of scores, amongst other things. However, the sheer number of articles found in our search suggests that the findings were fairly robust. The search period was constrained to the year of publication of the original NPI in 1979 through the year 2014. The original NPI was based on the DSM-III criteria for NPD. That criteria changed with the publication of the DSM-5 in mid-2013. The various incarnations of the DSM have both reflected and guided the conceptualization of narcissism, NPD, and other disorders. In 2013, the codification of the alternative model for personality disorders in the DSM-5 validated the concerns of trait researchers' reconceptualization of narcissism and saw a concurrent increase in the development of both trait-based and alternative measures of narcissism. Because of the time lag between the development of researchers' questions, their manuscript development, article submission, and article publication we thought it prudent to extend the search for studies using the NPI to include the calendar year 2014. By doing this, we were able to include the years in which studies measuring narcissism did so mainly with the NPI. The NPI is still in widespread usage but numerous efforts to supplant it exist [35]. This search was conducted in September 2016.

The PsychInfo and ABI-Inform databases were searched for the period since the seminal scale development article [4], using the keyword “narciss*” to capture all variations of “narcissism”, “narcissistic”, “narcissist”, “Narcissus”, etc. The PsychInfo database search resulted in 2552 peer-reviewed academic journal articles. The ABI-Inform search yielded 2323 articles. The Social Science Citation Index (SSCI) was searched for articles that cited one of the five seminal scale development and refinement articles [4,8,9,10,11]. This SSCI search resulted in 565, 259, 963, 389, and 404 articles, respectively, citing one of the aforementioned five seminal articles. The PsychInfo, ABI-Inform, and SSCI searches therefore resulted in 7455 possible articles. Of these articles, 6242 were duplicates and were eliminated resulting in 1213 unique articles for consideration.

**Fig 1:**
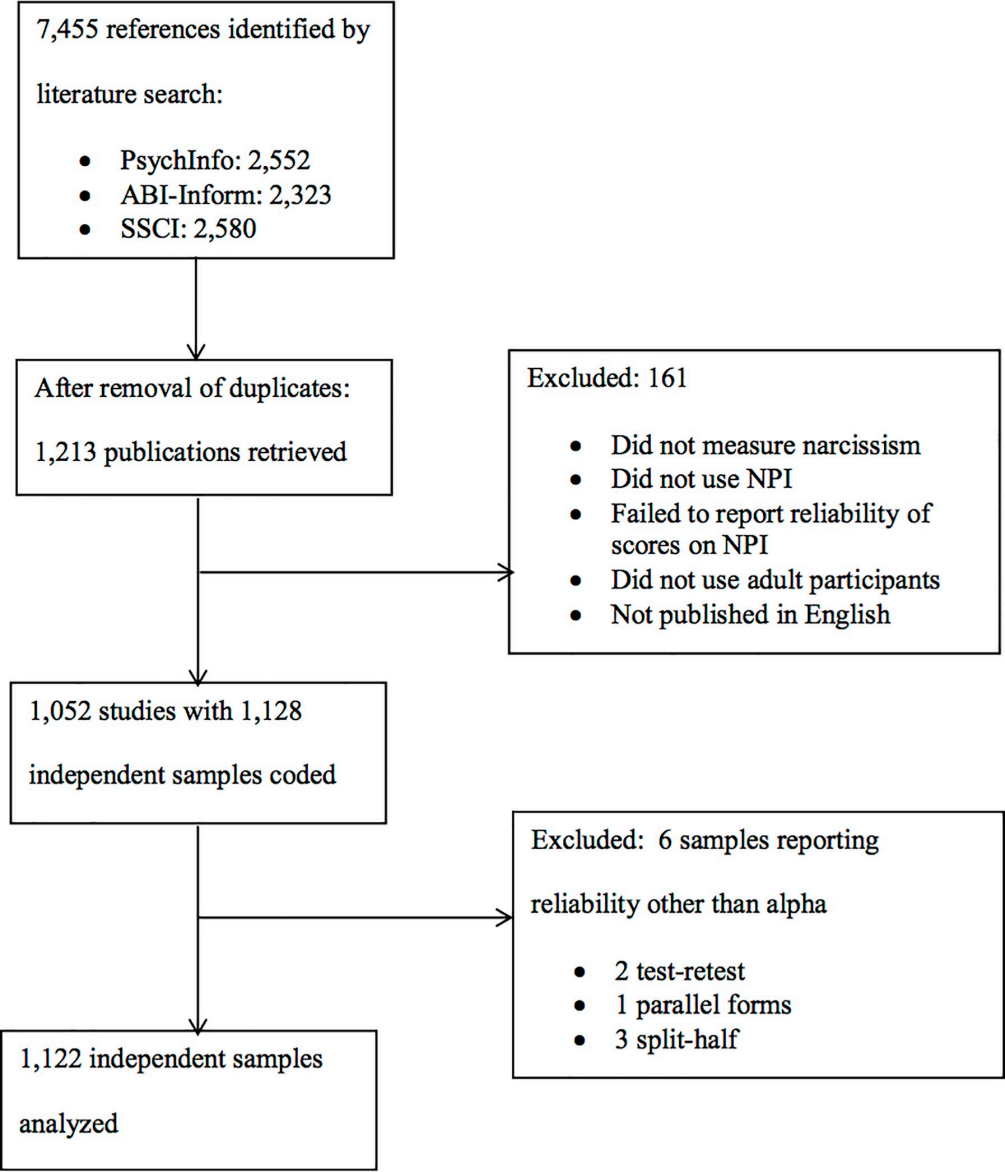
Flowchart for study selection. The steps below were used to identify and select studies for coding.

However, these three searches yielded many articles that were about narcissism but that did not actually measure narcissism, that measured narcissism with an instrument other than the NPI, that failed to report the reliability of scores on the NPI, did not use adult participants, or were not published in English. Each of these characteristics required the removal of the study from consideration and further-refined the sample to 1052 candidate studies. Because some studies reported on more than one independent sample of respondents, the number of independent samples rose to 1128. Psychometricians suggest that when vastly different forms of reliability (e.g. test-retest, parallel forms, Cronbach’s alpha) are reported, separate meta-analyses should be conducted [48,49]. Two samples reporting test-retest reliability, one reporting parallel forms reliability, and three others reporting split half reliability were therefore excluded from analysis reducing the number of independent sample to *k* = 1122. See the flowchart (Fig 1) of this study selection sequence. In contrast, the aforementioned recently published meta-analysis of various measures of narcissism [47] used only 124 samples. The independent sample was the unit of analysis in this study.

### Coding procedure

The NPI typically uses 40 self-report items in either a forced choice format or a Likert/Likert-type response format. Eqs 1 through 6 below [23] require the inclusion of the number of items and the sample size in the calculation of the variance (v) of the transformed reliability coefficient (T) for each independent sample (k) so we coded for these characteristics. The inverse of the variance of each transformed sample alpha is used as the weighting mechanism for each transformed alpha in the calculation of the population alpha. The goal of these transformations and calculations is to estimate the population reliability coefficient ($\hat{\rho }$) for all uses of the NPI.

With these thoughts in mind, we coded for the following: (1) reliability coefficient, (2) sample size, (3) number of items in scale, (4) type of response scale (coded as 0 = Likert / Likert-type or 1 = forced choice), (5) number of response options on the scale items (e.g. 2 for forced choice and typically 5 or 7 for Likert and Likert-type), (6) mean score on the scale, (7) standard deviation on the scale, (8) percentage of the sample who are non-White, (9) percentage of the sample who are female, (10) percentage of the sample who are of non-USA origin, (11) percentage of the sample who are college students, and (12) mean age of the sample. Study characteristics 1 through 3 were used for the computation of the transformed alpha as well as the variance of the reliability coefficients. Study characteristics 4 through 12 were used for moderator analyses described below.

### Coding agreement between raters

The candidate studies for inclusion were evenly split between three co-authors who read the articles for possible inclusion and coding. A fourth co-author cross-coded a random sub-sample of 15% of the studies coded by each of the three others. Disagreements were resolved by mutual agreement and overseen by a fifth co-author. The agreement indices calculated for each cross-coded study variable were the percentage agreement for every study characteristic and either the Cohen's kappa for categorical characteristics or the Pearson correlation for continuously scored characteristic. Overall, there was 85% agreement on the coding. For these agreement indices see Table 1.

**Table 1:** Agreement indices for coding of independent samples by two coders.

	Agreement indices		
	% agreement	kappa^a^	r^b^
1. Reliability coefficient	85	—	.86
2. Type of response scale	91	.88	—
3. Sample size	91	—	1.00
4. Number of items in scale	71	.79	—
5. Number of response options	71	.91	—
6. Mean score on scale	70	—	.98
7. Standard deviation on scale	76	—	.92
8. Percentage non-White	89	—	.98
9. Percentage female	78	—	.84
10. Percentage non-USA	90	—	.79
11. Percentage college students	98	—	.87
12. Mean age of sample	90	—	.85
Overall^c^	85	—	—

^a^ Cohen's kappa for categorically scored coding values.

^b^ Pearson correlation for continuously scored coding values.

^c^ Overall percentage agreement is not simply the average of the percentage agreements for the coded characteristics because not all primary studies reported every characteristic, thus the denominators for each characteristic's percentage agreement vary greatly.

### Data synthesis

The goal of a meta-analysis of coefficient alpha was to calculate the expected population alpha ($\hat{\rho }$) using sample alphas that were transformed to an effect size more normally distributed than the sample alphas and weighted by the inverse of the variance of the transformed alpha [23]. The variance weighting can be thought of as an indicator of the accuracy of alpha where alphas with smaller variances are more heavily weighted in the averaging procedure. Because alpha is almost never normally distributed, the first step was to transform the study effect *r* into T_i_ for scores in each independent sample *i* where α is either the KR-20 or Cronbach's alpha. The cube root of (1—r_α*i*_) transformed each study effect for non-normality as in Eq 1 below.

$$T_{i}={\left(1-r_{ai}\right)}^{1/3}$$ (1)

Then each sample effect (T_i_) was then weighted to compute the weighted mean transformed alpha [49].

$$\bar{T}=\frac{\Sigma (w_{i}T_{i})}{\Sigma w_{i}}$$ (2)

However, to compute $\bar{T}$. required the computation of the weighting factor (w) for each T_i_ in Eq 2 above. The weight (w) was the reciprocal of the variance of each transformed alpha

$$w_{i}=1/v_{i}$$ (3)

where the variance (v_i_) of the transformed alpha in sample *i* [42] in the equation below.

$$v_{i}=\frac{{18J}_{i}{\left(n_{i}-1)(1-r_{ai}\right)}^{2/3}}{{\left(J_{i}-1)(9n_{i}-11\right)}^{2}}$$ (4)

In order to calculate confidence intervals around $\bar{T}$., the variance of $\bar{T}$. was calculated as in the equation below.

v.=1Σwi (5)

The standard error of the mean = $\sqrt{v.}$ which allows for the calculation of confidence intervals around the mean transformed-and-weighted alpha as $\bar{T}.\pm z_{\alpha /2}\sqrt{v.}$ using the two-tailed critical value for z was calculated. Then $\bar{T}.$ was back-converted into α by using the equation below

$$\hat{\rho }=|1-{\bar{T}.}^{3}|.$$ (6)

Of course, the upper and lower confidence intervals were converted in a similar manner.

In order to test the null hypothesis that there were no significant differences in the study effects, the Q-statistic was used to test for homogeneity.

$$Q=\Sigma \frac{{\left(T_{i}-\bar{T}.\right)}^{2}}{v_{i}}$$ (7)

The Q-statistic is the ratio of between-sample variance to within-sample variance and is distributed as a chi-square with degrees of freedom equal to k-1 [50, 51]. Tests like those detailed above have been found to provide good control of Type I error for samples as small as 20 participants and for instruments with 20 to 40 items [23] like the NPI.

## Results

### Descriptive characteristics of the studies

We sought to estimate the population reliability coefficient for scores on the NPI and its sub-scales in samples that reported reliability as KR-20 or Cronbach’s alpha. In the independent samples using the overall NPI meta-analyzed here, the range of reliability coefficients (in the original metric) was from .61 [17] to .92 [18] with a mean of .82. The mean sample size and age of respondents was, 372.21 and 24.06, respectively. See Table 2 for these and other coded study characteristics for the overall NPI.

**Table 2:** Characteristics of coded studies for full-length Narcissistic Personality Inventory.

	k			
	Valid	Missing	Mean	SD
1. Reliability value	525	0	.82	.06
2. Number of items in scale	496	29	34.71	10.48
3. Type of response scale	426	99	.54	1.62
4. Number of points on scale	427	98	2.50	1.29
5. Mean score on scale	327	198	12.75	11.54
6. Standard deviation on scale	316	209	4.72	3.30
7. Percentage non-White	207	318	33.30	21.27
8. Percentage female	494	31	56.99	22.11
9. Percentage non-USA	74	451	93.07	23.80
10. Percentage college students	383	142	95.03	20.54
11. Mean age of sample	358	167	24.06	7.03
12. Sample size	524	1	372.21	1451.89

### Expected reliability coefficients

Using Eqs 1–6 above [23] required the transformation of each original reliability coefficient and then the calculation of the average transformed reliability. Each transformation was weighted by the reciprocal of the variance of the reliability because the variance is a proxy for the accuracy of the reliability estimate. Eq 4 above for the variance of alpha required three variables: (1) the alpha reliability value, (2) the number of items in the scale or sub-scale, and (3) the sample size on which the reliability was calculated. Numerous studies could not be used in these calculation because of the omission of the exact number of items used and/or the sample size.

#### Overall NPI

Because 29 of 525 samples failed to report the number of items in their version of the overall NPI (number of items ranged from 4 to 40) and one failed to report the sample size, the final and complete number of independent samples analyzed was *k* = 495 with an overall sample size of *n* = 195,038 (ranging from 40 to 25,849). The weighted transformed mean value [50] for this group of studies was $\bar{T}.$ = .5613 with lower and upper 95% confidence intervals of .5596 and .5630, respectively. After back-converting the average transformed value of $\bar{T}.$ and its confidence intervals, the resulting expected population reliability coefficient was ${\hat{\rho }}_{\alpha }$ = .8232 with lower and upper 95% confidence intervals of .8216 and .8247, respectively.

#### Raskin and colleagues’ original sub-scales

Authority is one of the original seven sub-scales of the NPI [4,8,11]. Unweighted alpha reliability indices ranged from .53 [52] to .90 [53] in 49 independent samples. For the 22 samples providing the necessary information, the expected population reliability coefficient for scores on the authority sub-scale was ${\hat{\rho }}_{\alpha }=.75$ with lower and upper 95% confidence intervals, respectively, of .74 and .76.

The unweighted alpha reliability indices for scores on the exhibitionism sub-scale ranged from .49 [15]to .86 [54,55] in 72 independent samples. For the 37 samples providing the necessary information, the expected population reliability coefficient was ${\hat{\rho }}_{\alpha }=.56$ with lower and upper 95% confidence intervals, respectively, of .56 and .57.

Scores on the superiority sub-scale resulted in unweighted alpha reliability indices that ranged from .41 [56] to .84 [57]in 45 independent samples. For the 24 samples providing the necessary information, the expected population reliability coefficient for scores on superiority was ${\hat{\rho }}_{\alpha }=.64$ with lower and upper 95% confidence intervals, respectively, of .63 and .65.

The measurement of entitlement resulted in unweighted alpha reliability indices that ranged from .31 [58] to .91 [59] in 79 independent samples. For the 55 samples providing the necessary information, the expected population reliability coefficient was ${\hat{\rho }}_{\alpha }=.67$ with lower and upper 95% confidence intervals, respectively, of .66 and .68.

Scores on the exploitativeness sub-scale showed unweighted alpha reliability indices that ranged from .30 [60] to .86 [61] in 52 independent samples. For the 31 samples providing the necessary information, the expected population reliability coefficient was ${\hat{\rho }}_{\alpha }=.67$ with lower and upper 95% confidence intervals, respectively, of .66 and .68.

Self-sufficiency scores resulted in unweighted alpha reliability indices that ranged from .30 [56] to .68 [61] in 32 independent samples. For the 15 samples providing the necessary information, the expected population reliability coefficient was ${\hat{\rho }}_{\alpha }=.48$ with lower and upper 95% confidence intervals, respectively, of .46 and .49.

Vanity is the last of the original sub-scales of the NPI [4,8,11]. Unweighted alpha reliability indices ranged from .50 [62] to .90 [63] in 29 independent samples. For the 13 samples providing the necessary information, the expected population reliability coefficient was ${\hat{\rho }}_{\alpha }=.67$ with lower and upper 95% confidence intervals, respectively, of .65 and .68.

#### Emmons’ revised sub-scales

Leadership/authority is one of the revised four sub-scales of the NPI [9,10]. Unweighted alpha reliability indices ranged from .63 [64] to .89 [65] on scores on this sub-scale in 78 independent samples. For the 37 samples providing the necessary information, the expected population reliability coefficient was ${\hat{\rho }}_{\alpha }=.76$ with lower and upper 95% confidence intervals, respectively, of .75 and .76.

Self-absorption/self-admiration scores resulted in unweighted alpha reliability indices that ranged from .60 [66] to .89 [67] in 35 independent samples. For the 13 samples providing the necessary information, the expected population reliability coefficient was ${\hat{\rho }}_{\alpha }=.65$ with lower and upper 95% confidence intervals, respectively, of .64 and .67.

Scores on the superiority/arrogance sub-scale of the NPI resulted in unweighted alpha reliability indices ranged from .41 [68] to .89 [69] in 34 independent samples. For the 12 samples providing the necessary information, the expected population reliability coefficient was ${\hat{\rho }}_{\alpha }=.60$ with lower and upper 95% confidence intervals, respectively, of .59 and .62.

Exploitativeness/entitlement is the last of the revised four sub-scales [9,10]of the NPI. Unweighted alpha reliability indices ranged from .12 [70] to .86 [9] in 82 independent samples. For the 39 samples providing the necessary information, the expected population reliability coefficient was ${\hat{\rho }}_{\alpha }=.66$ with lower and upper 95% confidence intervals, respectively, of .65 and .66. See Table 3 for these statistics.

**Table 3:** Meta-analytic results for NPI and its sub-scales.

						95% Confidence Intervals for $\hat{\rho }$	
Scale or subscale	k^a^	$\bar{T}$.	v.	$\sqrt{v.}$	$\hat{\rho }$	Lower	Upper
Full length NPI	495	.5612876	.0000007	.0008484	.8232	.8216	.8247
Raskin's original sub-scales:							
Authority	22	.6293796	.0000178	.0042255	.7507	.7407	.7604
Exhibitionism	37	.7600027	.0000032	.0017765	.5610	.5550	.5670
Superiority	24	.7101718	.0000171	.0041343	.6418	.6294	.6540
Entitlement	55	.6912584	.0000067	.0025903	.6696	.6624	.6769
Exploitativeness	31	.6890517	.0000118	.0034337	.6728	.6632	.6823
Self-sufficiency	15	.8060764	.0000185	.0043041	.4762	.4596	.4925
Vanity	13	.6935516	.0000460	.0067838	.6664	.6468	.6852
Emmons' revised sub-scales:							
Leadership / authority	37	.6222606	.0000042	.0020554	.7591	.7543	.7637
Self-absorption / self-admiration	13	.7039378	.0000264	.0051407	.6512	.6360	.6659
Superiority / arrogance	12	.7352266	.0000284	.0053365	.6026	.5854	.6193
Exploitativeness / entitlement	39	.6991871	.0000041	.0020323	.6582	.6523	.6640

Note: k = number of independent samples, $\bar{T}.$ = mean value of transformed alpha, v. = variance of mean transformed alpha, $\sqrt{v.}$ = standard error of mean, $\hat{\rho }$ = expected population value of alpha

^a^ Number of studies reporting sub-scale alpha greatly reduced because not all provided number of items, sample size, or both

#### Tests of homogeneity of effects

The Q-statistic was calculated to determine if the distribution of effects on the overall NPI was homogenous. The Q-statistic, distributed as a chi-square, was equal to 1,955,625.90 with 494 degrees of freedom (p < .001). Thus, there were statistically significant variations in the distribution of individual reliability coefficients that necessitated moderator analyses. Because no single study reported all coded characteristics, moderator tests conducted via multiple regression, which makes use of listwise deletion, would have reduced the sample to nil. Therefore, simple bivariate correlations were computed to ascertain if any coded characteristics were related to the reliability coefficients in the sample of coded studies using pairwise deletion.

The results of the bivariate analyses follow. Consistent with the Spearman-Brown prophecy formula, the number of items used in the NPI was significantly related (*r* = .44, *p* < .001, *k* = 496) to the reliability of scores indicating that longer scales resulted in higher reliability. Other coded characteristics significantly related to the reliability of scores on the overall NPI were type of response scale (coded as 0 = Likert / Likert-type or 1 = forced choice; point-biserial *r* = -.18, *p* < .001, *k* = 496). Thus, Likert or Likert-type scales resulted in higher reliability than did the original forced choice style. The mean score on the scale was related to the reliability of those scores (*r* = .19, *p* < .01, *k* = 327) such that as the mean score on a scale increased so did the reliability of scores on that scale. Additionally, the standard deviation of scores on the scale was related to the reliability of those scores (*r* = .28, *p* < .001, *k* = 316) in that a greater spread of scores on the scale was associated with a higher reliability of those scores. Lastly, the percentage of the sample who were female was related to the reliability of the scores for the samples (*r* = .10, *p* < .05, *k* = 494). Therefore, samples with more females provided better reliability of scores on the overall NPI. Neither the percentages of each sample who were non-White, who were of non-USA origin, and who were college students nor the sample size or the mean age of the sample were related to NPI reliability.

In an effort at finding a multivariate predictive model that would explain some of the variance in the sample reliability coefficients we used meta-regression with the five coded characteristics reported above as being statistically significant. This regression model allowed for the simultaneous examination of these variables in their contribution to incremental validity above and beyond each other. Because regression uses listwise deletion, the sample size was reduced to 244 independent samples. The overall equation resulted in *F* (*df*_1_ = 5, *df*_2_ = 238) = 33.50 (*p* < .001) that explained 41% of the variance in sample alpha. Of the five regression coefficients, number of items in the scale, standard deviation of scores on the scale, and type of response scale were statistically significant predictors whereas mean score on the scale and percentage of the sample who were female were not. The individual effect sizes for the three significant predictors computed as the squared semi-partial correlation coefficient were, respectively, .22, .02, and .15. See Table 4 for the above results.

**Table 4:** Meta-regression of reliability coefficients on three coded sample characteristics ^a^.

	Reliability coefficient as criterion					
				95% confidence intervals		Effect
Variable	B	s.e.	β	Lower	Upper	size ^b^
Constant	.777	.014		.750	.805	
Number of items	.003	.000	.568***	.002	.004	.220
Mean score on scale	-.001	.000	-.164	-.002	.000	.008
SD of scores on scale	.005	.002	.275**	.001	.008	.017
Percentage female	.000	.000	.087	.000	.000	.007
Type of response scale ^c^	-.016	.002	-.443***	-.019	-.012	.150
F-score _(df1, df2)_	33.50_(5, 238)_***					
R^2^	.413					
adjusted R^2^	.401					

^a^ n = 244.

^b^ Squared semi-partial correlation.

^c^ Coded as 0 = forced choice, 1 = Likert or Likert-type.

** p < .01.

*** p < .001.

The Q-statistic was not calculated on the sub-scales. Many samples did not provide the number of items in their sub-scale, the sample size, or both so the various number of samples ranged from only *k* = 12 for the revised sub-scale of superiority/arrogance [9,10] to *k* = 55 for the original entitlement sub-scale [4,8,11]. Because analysis on such few observations would lack statistical power, no moderator tests were implemented for the sub-scales.

## Discussion

A reliability generalization (RG) study allows researchers to determine the expected population coefficient alpha for scores on an instrument as well as the study, scale, and sample characteristics associated with the reliability of scores in independent samples of respondents. The expected population coefficient alpha of internal consistency reliability meta-analytically determined here for scores on the overall Narcissistic Personality Inventory was .82. This population value had narrow confidence intervals and was based upon almost 500 independent samples with almost 200,000 participants. The expected population value for alpha on the sub-scales of the NPI varied greatly. All in all, the sub-scale population alphas were much weaker than the overall NPI alpha.

There was significant heterogeneity in the sample reliability values for the full-length NPI that was shared with some of the coded characteristics. Consistent with the Spearman-Brown formula and despite not using parallel items the reliability of the scale improved with the number of items used in the scale. Additionally, score reliability was higher as the mean score on the scale increased. Observed scores are an approximation of latent traits so for an item response theory examination of the measurement precision of the sub-scales of the NPI across all levels of the latent trait see the work of Grosz et al. [16]. Because the variance of test scores is part of the formula for Cronbach’s alpha and KR-20 it is no surprise that more reliable scores were obtained when the standard deviation for scores on the scale was larger. Additionally, the use of Likert or Likert-type scales resulted in higher reliability than when data were collected with the original forced choice response scale which is consistent with past research [16]. In subsequent regression analysis with a listwise reduced sample, three characteristics remained significant and they alone explained over 41% of the variability in alpha. It is noteworthy that 354 samples reported the reliability of scores as Cronbach’s alpha but used a forced choice instrument. Technically, the reliability index was a KR-20. However, the KR-20 is a mathematical simplification of alpha so it is acceptable, if not altogether precise, to use the terms interchangeably.

### Limitations

Of course, no discussion of findings related to coefficient alpha would be complete without a critical overview of the limitations of alpha as an indicator of score reliability. At least three sets of authors [71, 72, 73] have provided consistent acknowledgment that alpha is the lower bound of reliability and that it requires that items be at least tau equivalent. Additionally, for the Spearman-Brown formula to be truly effective for determining the length of a scale necessary to reach a certain level of reliability the rule is even stricter such that the items must be parallel. Items in the NPI are neither parallel nor tau equivalent. Compounding this is the fact that the NPI is decidedly not uni-dimensional [13] and scores on the sub-scales are sometimes offsetting so combining sub-scales comprised of purposefully non-parallel nor tau equivalent items to improve the reliability of scores is ill-advised. Primary study authors should consider other measures of reliability like omega when appropriate and at least acknowledge the shortcomings of alpha when used.

Additionally, it should be noted that most research using the NPI made use of college students (i.e. 95% of respondents). Such a narrowly defined set of respondents likely limits the generalizability of research on the validity of the NPI. However, it is noteworthy that neither the average age of the samples nor the percentage of a sample who were college students was related to the reliability of scores on the NPI.

### Implications

These results have some implications. For those psychometricians seeking to validate Big Five facet-based measures of narcissism, the reliability of scores on the overall NPI is vital. The often misused cutoffs for reliability indicate that in “applied settings where important decisions are made with respect to specific test scores, a reliability of .90 is the minimum that should be tolerated, and a reliability of .95 should be considered the desirable standard” (p. 226) [74]. With these thoughts in mind, a population value of .82 for alpha as an approximation of reliability falls short of even the least rigid of Nunnally’s two cutoffs [74] and the population values for the various sub-scales are woefully inadequate. The original version [4] of the NPI was developed based upon the clinical diagnostic criteria for NPD in the DSM-III [6]. However, the criteria for diagnosing NPD has since changed (see the DSM-5) and the NPI fell out of favor for its diagnosis. Separate attempts at refining the NPI [14] for the measurement of sub-clinical narcissism as well as the numerous attempts at the dimensional measurement of NPD using facet scores on the Big Five traits look promising. As a multi-factor instrument, the NPI is not without its shortcomings, but it continues to be the most used instrument for measuring sub-clinical non-pathological narcissism. Further refinement of the NPI will likely parallel interest in expanding the study of narcissism amongst personality researchers as a component of the Dark Triad [3] and the newly developed Dark Tetrad [75] as well as a general increase in the study of the dark side of personality [76].

## Supporting information

S1 Checklist Steps in this systematic review. (DOC)

S1 Dataset Dataset for coded studies. (CSV)

S1 Coding Set Coded study variables. (DOC)
