# Supplementary material for: Meta-analysis of coefficient alpha for scores on the Narcissistic Personality Inventory
Source: PLoS One. 2018 Dec 4;13(12):e0208331. doi: 10.1371/journal.pone.0208331 (PMC6279043; doi:10.1371/journal.pone.0208331)
Supplement: S1 Coding Set — (DOC) [file pone.0208331.s003.doc]

| SubscaleName | Reliability Score | Reliability Type ^a^ | Number of items in scale | Type of response scale ^b^ | # of response options | Sample size | Mean score on scale | StD on scale | % of sample ethnic minority | % of sample female | % of sample international | % of sample college students | Mean age of sample | Comments |
| --- | --- | --- | --- | --- | --- | --- | --- | --- | --- | --- | --- | --- | --- | --- |
| Overall NPI = **0** |  |  |  |  |  |  |  |  |  |  |  |  |  |  |
| Authority = **1** |  |  |  |  |  |  |  |  |  |  |  |  |  |  |
| Exhibitionism = **2** |  |  |  |  |  |  |  |  |  |  |  |  |  |  |
| Superiority = **3** |  |  |  |  |  |  |  |  |  |  |  |  |  |  |
| Entitlement = **4** |  |  |  |  |  |  |  |  |  |  |  |  |  |  |
| Exploitativeness = **5** |  |  |  |  |  |  |  |  |  |  |  |  |  |  |
| Self-sufficiency = **6** |  |  |  |  |  |  |  |  |  |  |  |  |  |  |
| Vanity = **7** |  |  |  |  |  |  |  |  |  |  |  |  |  |  |
| Leadership / Authority = **8** |  |  |  |  |  |  |  |  |  |  |  |  |  |  |
| Self-absorp. / Self-admir. = **9** |  |  |  |  |  |  |  |  |  |  |  |  |  |  |
| Superiority / Arrogance = **10** |  |  |  |  |  |  |  |  |  |  |  |  |  |  |
| Exploitat. / Entitlement = **11** |  |  |  |  |  |  |  |  |  |  |  |  |  |  |
| Demographic variables measured: | | | | | | | | | | | | | | |
| Organizational variables measured: | | | | | | | | | | | | | | |
| Psychological variables measured: | | | | | | | | | | | | | | |
| Comments: | | | | | | | | | | | | | | |

^a^ 1 = Cronbach’s alpha, 2 = KR-20, 3 = test-retest, 4 = parallel forms, 5 = split half, 6 = other

^b^ 1 = Likert, 2 = Likert type, 3 = Numeric response, 4 = Graphic rating, 5 = Semantic differential, 6 = Forced choice
